# An Echocardiographic Insight Into Post-COVID-19 Symptoms

**DOI:** 10.7759/cureus.38039

**Published:** 2023-04-24

**Authors:** Rui Flores, Olga Pires, Joana Alves, Vítor Hugo Pereira

**Affiliations:** 1 Cardiology, Hospital de Braga, Braga, PRT; 2 Internal Medicine, Hospital de Braga, Braga, PRT; 3 Infectious Disease, Hospital of Braga, Braga, PRT

**Keywords:** right heart failure, pulmonary and cardiac pathophysiology, tako-tsubo cardiomyopathy (ttc), heart failure, covid 19

## Abstract

Introduction and objectives: Severe acute respiratory syndrome coronavirus-2 (SARS-CoV-2) infection has frequent acute cardiovascular manifestations, but long-term sequelae are yet to be described. Our main objective is to describe the echocardiographic findings of patients with a previous SARS-CoV-2 infection.

Methods: A single-center prospective study was conducted. Patients who tested positive for SARS-CoV-2 were selected and submitted to a transthoracic echocardiogram six months after infection. A complete echocardiographic assessment was performed, including tissue Doppler, E/E' ratio, and ventricular longitudinal strain. Patients were divided into two subgroups according to their need for admission to the ICU.

Results: A total of 88 patients were enrolled. The mean values and respective standard deviations of the echocardiographic parameters were as follows: left ventricular ejection fraction 60.8 ± 5.9%; left ventricular longitudinal strain 17.9 ± 3.6%; tricuspid annular plane systolic excursion 22.1 ± 3.6 mm; a longitudinal strain of the free wall of the right ventricle 19.0 ± 6.0%. We found no statistically significant differences between subgroups.

Conclusions: At the six-month follow-up, we found no significant impact of past SARS-CoV-2 infection on the heart using echocardiography parameters.

## Introduction

Since the first cases were described in Wuhan, China in late 2019, the severe acute respiratory syndrome coronavirus-2 (SARS-CoV-2) infection spread worldwide in just a few months, instigating fear and anxiety, menacing international borders, and raising awareness about health implications such as cardiovascular disease [1-3]. The exact mechanism by which this association occurs is not fully understood, advocating the need for more studies on the cardiovascular manifestations and/or complications of SARS-CoV-2 infection.

Studies using MRI show the presence of myocardial edema and right ventricular dysfunction during and after a SARS-CoV-2 infection [4]. This cardiovascular involvement may be largely underestimated [5]. The incidence of acute myocardial infarction is also not negligible [6]. Whether this cardiac involvement could have any long-term implications is yet unknown. Recent studies have shown that a significant proportion of patients with a past SARS-CoV-2 infection develop symptoms of fatigue and dyspnea [7]. We hypothesized that these symptoms could be related to ventricular dysfunction. Our main objective is to describe the echocardiographic findings of patients with a previous SARS-CoV-2 infection.

## Materials and methods

We conducted a prospective study at the Hospital of Braga. The project was approved by the institutional ethics committee (approval no. 210/2020). All patients who tested positive for SARS-CoV-2 between March and April 2020 were included in the study, whether they were hospitalized or not. For hospitalized patients, discharge was based on clinical and laboratory criteria, which included: (1) absence of respiratory failure or symptoms affecting the quality of life; (2) absence of fever for at least three days; (3) two consecutive negative tests (24 hours apart) by polymerase chain reaction (PCR); (4) laboratory tests without evidence of systemic inflammatory syndrome; and (5) absence of another clinical situation that justified prolonged hospitalization. All patients were referred for a transthoracic echocardiogram (TTE) about six months after discharge. Informed consent was obtained from all patients. We excluded patients who died during hospitalization or less than six months after hospital discharge. Patients under 18 years of age and patients with a bad echocardiographic window were also excluded. All data were collected in accordance with relevant guidelines and regulations.

All TTEs were ECG-gated (Vivid E95, GE Healthcare, Chicago, IL, USA) and involved a routine cardiac assessment (including a trial and ventricular dimensions, ventricular ejection fraction, and tricuspid annular plane systolic excursion), complemented with myocardial velocity assessment by tissue Doppler, the E/E' ratio, and ventricular longitudinal strain. The evaluation of left ventricular longitudinal strain was performed using the speckle tracking method and by combining the strain obtained in the apical two, three, and four chambers. The right ventricular longitudinal strain was obtained using the speckle tracking method of the free wall of the right ventricle in an apical four-chamber configuration. The demographic analysis was made based on the mean ± standard deviation, and range of values. Patients were divided into two subgroups: with and without the need for admission to an ICU. The previously mentioned parameters were compared between the two subgroups. Differences between subgroups were made using the T-test for independent samples. A p-value of < 0.05 was considered statistically significant.

## Results

Demographics

A total of 88 out of 198 patients were enrolled after applying the inclusion and exclusion criteria. Of the 110 excluded patients, 47 (42.7%) died during hospitalization (more than 80% were more than 80 years of age) mostly due to severe respiratory insufficiency; four patients died during the follow-up period (one patient died due to an acute myocardial infarction and three died due to pulmonary infections); 17 patients were less than 18 years of age; 12 patients had a bad echocardiographic window that impaired correct quantitative data collection; and 30 patients refused to give their informed consent. Eighty-three patients (94.3%) were hospitalized. The mean age was 62 ± 13 years old (28-96 years old). Most patients were men (n=50, 56.8%). Thirteen patients were admitted to ICUs and required invasive ventilation during hospitalization (14.8%). Table 1 illustrates the patient demographics.

**Table 1:** Patient demographics and echocardiographic data PSAP: Pulmonary systolic arterial pressure; TAPSE: Tricuspid annular plane systolic excursion

Patient demographics (n=88)	
Age (mean ± standard deviation, years)	62 ± 13
Male/Female (n)	50/38
Requiring hospitalization (n, %)	83 (94.3%)
Requiring admission to ICU	13 (14.8%)
Echocardiographic data* (n = 88 patients)	
Left atrium diameter	38.6 ± 4.7 mm
Left atrium area	19.5 ± 4.1 cm2
Left ventricular ejection fraction	60.8 ± 5.9%
Left ventricular longitudinal strain	- 17.9 ± 3.6%
Lateral E’	9.17 ± 2.7 cm/s
Septal E'	7.1 ± 1.8 cm/s
Basal diameter of the right ventricle	32.6 ± 5.0 mm
TAPSE	22.1 ± 3.6 mm
Right ventricular longitudinal strain	- 19.0 ± 6.0%
Estimated PSAP	31.1 ± 8.9 mmHg

Echocardiographic Findings

**Figure 1:**
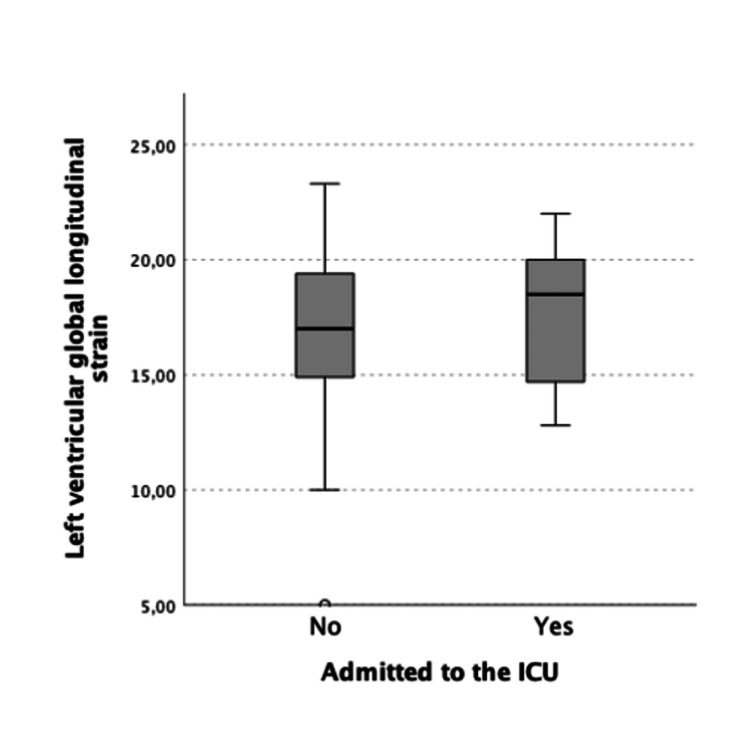
Median and interquartile range of left ventricular global longitudinal strain in patients admitted and not admitted to the ICU

**Figure 2:**
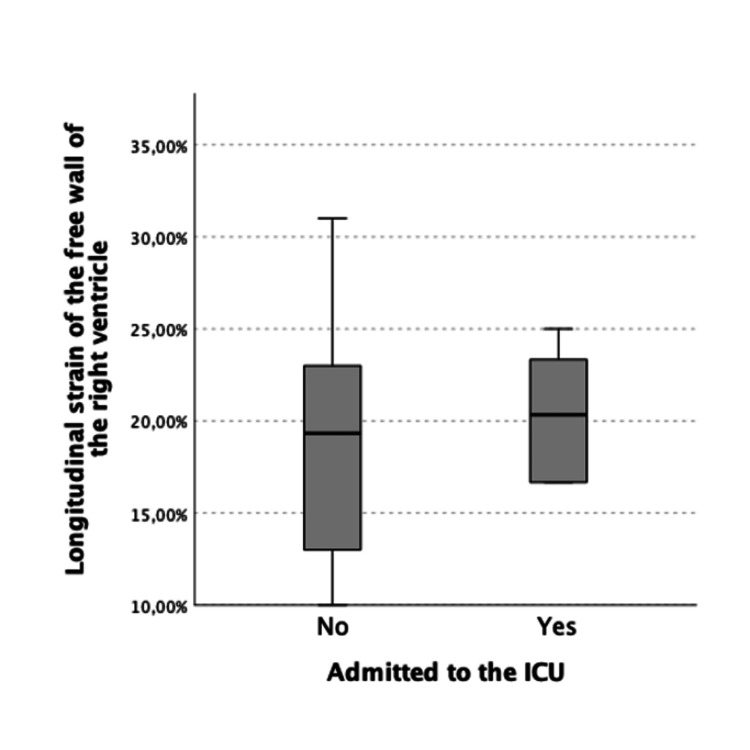
Median and interquartile range of longitudinal strain of the free wall of the right ventricle in patients admitted and not admitted to the ICU

Table 1 lists the echocardiographic findings. The mean values and respective standard deviations of the echocardiographic parameters were as follows: diameter of the left atrium 38.6 ± 4.7 mm; left atrium area 19.5 ± 4.1 cm2; left ventricular ejection fraction (LVEF) 60.8 ± 5.9%; left ventricular longitudinal strain 17.9 ± 3.6%; lateral E’ 9.17 ± 2.7 cm/s; septal E' 7.1 ± 1.8 cm/s; basal diameter of the right ventricle 32.6 ± 5.0 mm (20 to 46 mm); tricuspid annular plane systolic excursion (TAPSE) 22.1 ± 3.6 mm (17 to 32 mm); the longitudinal strain of the free wall of the right ventricle 19.0 ± 6.0%; estimated pulmonary artery systolic pressure 31.1 ± 8.9 mmHg. Three patients (3.4%) showed left ventricular dysfunction (LVEF <50%). We found no statistically significant differences between the subgroup of patients admitted to the ICU and the remainder in relation to any of the determined echocardiographic parameters (Table 2; Figures 1-2).

**Table 2:** Echocardiographic differences between patients admitted and not admitted to ICUs PSAP: Pulmonary systolic arterial pressure; TAPSE: Tricuspid annular plane systolic excursion

	ICU-admitted patients (n = 13)	Patients not admitted to ICU (n = 75)	p-value
Left atrium diameter (mm)	41.0	38.4	0.39
Left atrium area (cm2)	21.6	19.2	0.66
Left ventricular ejection fraction (%)	62.9	60.5	0.74
Left ventricular longitudinal strain (%)	- 17.8	- 17.0	0.93
Lateral E’ (cm/s)	7.4	9.4	0.02
Septal E'	6.4	7.2	0.16
Basal diameter of the right ventricle (mm)	35.2	32.1	0.22
TAPSE (mm)	21.1	22.2	0.15
Right ventricular longitudinal strain (%)	- 20.4	- 18.8	0.22
Estimated PSAP (mmHg)	31.2	31.1	0.33

## Discussion

Six months after a SARS-CoV-2 infection, our echocardiographic findings showed globally preserved left and right ventricular systolic function. There were no statistically significant differences between critically ill and mild-to-moderately ill patients.

Symptoms of tiredness and fatigue are frequent among patients who have experienced a SARS-CoV-2 infection. Recently, Huang et al. described a cohort of 1733 patients after a COVID-19 infection [7]. About six months after the acute phase, most patients had symptoms of dyspnea, fatigue, or depression [7]. There was a higher incidence of cardiorespiratory symptoms in the subgroup that was admitted to ICUs [7].

The acute cardiac injury induced by SARS-CoV-2 is well described by several authors using high-sensitivity troponin and cardiac MRI (c-MRI) [1,2,8,9]. For instance, Arcari et al. studied the impact of cardiac biomarkers on the outcomes of patients with COVID-19 pneumonia and concluded that the myocardial involvement of the virus is common and has a great impact on prognosis [10-13]. Troponin levels are frequently elevated in critically ill patients and may be more related to systemic disease (severe hypoxia, multiorgan failure, severe metabolic acidosis) than to virus-related disease itself [11]. Despite being associated with a worse acute prognosis, the long-term implications of this myocardial involvement are still unclear [9-14]. According to our data, this acute myocardial injury does not appear to be related to long-term impaired ventricular function.

Puntmann et al. noticed frequent myocardial involvement in recent COVID-19 illness by c-MRI [15]. Huang et al. concluded that myocardial edema and late gadolinium enhancement evaluated by c-MRI were frequent after SARS-CoV-2 infection, which may be underestimated by an echocardiographic evaluation [4]. Nonetheless, results from c-MRI should be regarded carefully in the subgroup of athletes because this population may present certain late gadolinium enhancement areas that are the result of ventricular remodeling due to exercise (and not myocarditis) [16]. Accordingly, due to its low specificity, myocardial edema evaluated through T2 mapping sequences may overestimate the cardiac involvement of the SARS-CoV-2 infection [17]. Breitbart et al. found a low incidence (2%) of myocarditis in these patients if strict criteria were used [17].

The recent study published by Huang et al. shared knowledge of the magnetic resonance findings of patients who had recovered from COVID-19 [4]. They concluded that the incidence of myocardial edema, fibrosis, and compromised right ventricular function was relatively high. Nonetheless, long-term assessment of cardiac imaging was not in the scope of the study, so these cardiac abnormalities may just be transient in the spectrum of COVID-19 disease [4].

The exact mechanisms by which COVID-19 impacts the heart are unknown [18]; nonetheless, recent studies have shed light on possible explanations, including localized and systemic inflammation, cytokine-mediated myocardial injury, thromboembolic phenomena, and stress-induced damage [11,12,19]. Certain angiotensin-converting enzyme II (ACE II) polymorphisms may explain different susceptibilities to severe disease [11]. Luciani et al. described a case where they explored the potential influence of the bacille Calmette-Guérin (BCG) vaccine in the development of an immune response to SARS-CoV-2. In their case report, the patient simultaneously developed severe SARS-CoV-2 pneumonia and tuberculosis due to a possible loss of BCG immunity [20].

Several studies have raised concerns about the increased thromboembolic risk of patients with COVID-19, particularly the increased incidence of pulmonary thromboembolism in the acute phase [21,22]. Therefore, the incidence of right ventricular dysfunction and pulmonary hypertension would be expected to be high. Liu et al. showed that a significant proportion of patients admitted to the ICU exhibited right ventricular dysfunction, and the prevalence of right ventricular dysfunction was higher than left ventricular dysfunction [23]. Our study was focused on long-term echocardiographic findings, so on the contrary, mean values of TAPSE and right ventricular longitudinal strain were within normal values, which partially contradicts this theory or, at least, indicates that pulmonary emboli are either of low risk or associated with microthrombosis [24-26]. One can presume that the lack of significant right ventricular dilation or pulmonary hypertension can be explained by our sample size. Another possible assumption is that the recovery from a pulmonary embolism after six months is favorable.

Our study had several limitations. First, most patients did not have an echocardiogram before admission or even during hospitalization, which prevented a comparative study. Furthermore, some indirect measures, such as cardiac output, were not explored. Second, the characteristics of the patients excluded from the study were not explored, which created a non-homogeneous population. Third, our evaluation was mostly centered on the echocardiographic findings of COVID-19, which can largely underestimate the cardiac involvement compared to MRI [4]. Finally, our sample is relatively small.

## Conclusions

In conclusion, our data lead us to conclude that long-term symptoms may be related to pulmonary, and less likely, cardiac involvement. The absence of relevant echocardiographic findings six months after COVID-19 infection may increase our attention to pulmonary vascular complications or potentially irreversible lesions of the lung parenchyma. Overall, further studies are needed to clarify the cause of these symptoms and assess the impact on long-term events. It is important to create prospective studies with larger populations to understand whether our conclusions are reproducible.
